# Resistance to malaria in humans: the impact of strong, recent selection

**DOI:** 10.1186/1475-2875-11-349

**Published:** 2012-10-22

**Authors:** Philip W Hedrick

**Affiliations:** 1 School of Life Sciences, Arizona State University, Tempe, AZ, 85287, USA

**Keywords:** Age of allele, Duffy, Epistasis, Sickle cell, Thalassaemia

## Abstract

Malaria is one of the leading causes of death worldwide and has been suggested as the most potent type of selection in humans in recent millennia. As a result, genes involved in malaria resistance are excellent examples of recent, strong selection. In 1949, Haldane initially suggested that infectious disease could be a strong selective force in human populations. Evidence for the strong selective effect of malaria resistance includes the high frequency of a number of detrimental genetic diseases caused by the pleiotropic effects of these malaria resistance variants, many of which are “loss of function” mutants. Evidence that this selection is recent comes from the genetic dating of the age of a number of these malaria resistant alleles to less than 5,000 years before the present, generally much more recent than other human genetic variants. An approach to estimate selection coefficients from contemporary case–control data is presented. In the situations described here, selection is much greater than 1%, significantly higher than generally observed for other human genetic variation. With these selection coefficients, predictions are generated about the joint change of alleles *S* and *C* at the *β*-globin locus, and for *α*-thalassaemia haplotypes and *S*, variants that are unlinked but exhibit epistasis. Population genetics can be used to determine the amount and pattern of selection in the past and predict selection in the future for other malaria resistance variants as they are discovered.

## Background

Malaria is one of the leading causes of death worldwide and has been suggested as the most potent type of selection in humans in recent millennia [1]. As a result, genes involved in malaria resistance are excellent examples of recent, strong selection. Perhaps best known is the sickle cell haemoglobin variant, which is often used as an example of heterozygote advantage. In addition, G6PD deficiency illustrates strong selection at an X-linked locus, *β*-globin variants *S*, *C*, and *E* and G6PD deficiency variants *A*-, *Med*, and *Mahidol* show how selective differences can be the result of a single-nucleotide change. Further, *HLA-B53* illustrates how gene conversion can result in an adaptive allele, and *α*-thalassaemia shows how selection can operate on loci that have different copy numbers.

Haldane, known as one of the three founders of population genetics, is often recognized with first suggesting that disease could be an important evolutionary force in humans. Although his general review [2] is often cited for this concept, this hypothesis was presented in more detail in 1948 [3] where he suggested that *β*-thalassaemia heterozygotes had an increased fitness in the presence of malaria. Therefore, citation of [3] seems more correct for the hypothesis that malaria resistance in humans might be genetically determined and evolutionarily significant [4-6].

The first generally recognized evidence for genetic resistance to malaria in humans was in 1954 [7] for sickle-cell haemoglobin heterozygotes *AS*. Overall, the “malaria hypothesis” of Haldane that some human diseases such as thalassaemia are polymorphisms and provide heterozygote advantage because of the trade-offs between the advantages of resistance to malaria and negative effects due to the disease, is now widely accepted but the exact means of disease resistance have often been difficult to elucidate.

As documentation for the contemporary influence of malaria, in 2010 there were 216 million clinical cases and an estimated 863,000 deaths from malaria [8], making it one of the leading causes of death worldwide. Although these levels have declined globally 25% since 2000, another analysis estimated that the annual mortality may be much higher than this level at 1.24 million [9].

The impact of malaria is thought to have increased between 10,000 and 5,000 years ago when there were the beginnings of agriculture and consequently more human settlements. During this period, the numbers of both the human population and the mosquito vector increased, resulting in higher spread of malaria [10]. Recent molecular studies suggest that malaria in humans from *Plasmodium falciparum* may have originated from gorillas [11,12]. Using these data, an initial timeline for the origin of *P. falciparum* as a human pathogen suggests that it may be more recent than previously thought [13].

The past geographic extent of malaria and the distribution of malaria resistance variants broadly correspond [14-16]. For example, *P. falciparum* is found across Africa and Asia, as are the variants of haemoglobin and G6PD that provide malaria resistance. In regions of high endemic malaria, such as sub-Saharan tropical Africa and lowland Melanesia, there are often several variants. In contrast, variants do not exist in areas without past malaria, with the exception of ancestry from new immigrants [17]. To illustrate, the common malaria resistant alleles are not present in New World natives [18], presumably because their ancestors were unexposed to malaria and malaria only came to the Americas during the transatlantic slave trade between the 16^th^ and 19^th^ centuries [19]. Further, microgeographic variation of *β*-thalassaemia in Sardinia and the past presence of malaria are concordant [20] and *α*-thalassaemia and *β*-thalassaemia variation in Melanesia is associated with malaria presence [21,22].

Some resistance alleles for malaria are distinctive and others are very general. To illustrate, geographically separated Duffy alleles in Papua New Guinea and Africa result from changes at the same exact genomic location, 33 nucleotides upstream from the start codon. Similarly, the *β*-globin malaria resistance alleles *S* and *C* occur from different changes at the same codon. In contrast, there are many changes that modify levels of expression and provide malaria resistance for G6PD deficiency, *α*-thalassaemia, and *β*-thalassaemia.

It is significant that malaria resistance genes are often extremely variable, for example, the malaria resistance genes ABO, G6PD, *HLA*, *α*-globin, and *β*-globin, are some of the most variable human genes. Such variation might be because disease resistance genes have high amounts of standing variation or because these genes have high mutation rates and produce new adaptive alleles very quickly.

There are several current first-rate reviews of the mechanisms of malaria resistance and evaluations of the malaria resistance genes [1,23-26] and in an extensive recent review, many aspects of the population genetics of malaria resistance were examined [6]. Therefore, the focus here will be on the data indicating the very strong and recent selection for malaria resistance in humans. In addition, it will be shown how the expected change when there is strong selection for malaria resistance variants can be predicted with population genetics models.

## Strength of selection

### Pleiotropic disease effects

Strong selection for malaria resistance has increased, due to pleiotropic effects, the frequency of genetic diseases, such as G6PD deficiency, the thalassaemias, and ovalocytosis. For example, it has been suggested that this has caused a worldwide problem and has resulted in 300,000 newborns each year with haemoglobin maladies [27]. These pleiotropic effects explain why such disorders are the most common genetic diseases [28]. It is also possible that some other diseases may be in high frequency because of the pleiotropic effects of malaria resistance variants [1].

Around 400 million people (worldwide prevalence of 4.9%) are affected with G6PD deficiency, the most common enzymopathy in humans [29]. Even though people with G6PD deficiency are often asymptomatic, various factors that result in oxidative stress can cause disease episodes. To illustrate, fava beans can cause severe haemolytic anaemia (favism) for individuals with the *Med* G6PD deficiency.

Perhaps related to the high frequency of diseases related to genes conferring resistance to malaria is that many of resistance variants, such as G6PD deficiency, the thalassaemias, sickle-cell anaemia, and ovalocytosis, are “loss of function” mutants and lead to reduced expression or altered gene product. Sometimes these variants result in disease because of their pleiotropic effects. Or, in other situations, there is no cost to the change in expression or gene product, as appears to be the case for the Duffy “null” variants and for *β*-globin gene allele *C*.

### Age of selective variants

Many malaria resistance genetic variants appear to be recent polymorphisms and have been generated in the last 5,000 to 10,000 years or less (see Table 1 and below). These variants appear to date to the time when severe malaria first became an important human evolutionary factor. From this, one can surmise that they were not malaria resistance alleles in ancestral species, i.e., they are not trans-specific polymorphisms, and that they most likely were the result of standing variation or generated by new mutations. Homologous resistance alleles are not generally found in other primates [30-32] or if they are, the variants are different from human variants [33].

**Table 1:** Estimated age of malaria resistance variants in years (assuming a 25-year generation length) and the selection coefficient (estimated or assumed), --- indicates no selection (neutrality) is assumed

**Gene**			
**allele**	**Age**	**Selection coefficient**	**Reference**
*β* -globin			
*S*	1,440	0.152	[34]
*C*	2,810	0.06	[36]
*E*	2,510	0.079	[39]
G6PD			
*A-*	6,360	0.044	[40]
*A-*	1,000	0.25	[41]
*Med*	3,330	0.034	[40]
*Mahidol*	1,580	0.23	[42]
HLA-B			
*B53*	2,150	0.041	[6]
Duffy			
*ES* (null)	33,000	---	[49]
*ES* (null)	10,000	---	[50]

The age of *S* alleles has been estimated from the amount of association (linkage disequilibrium) in sequence near the *HBB* locus. To illustrate, all samples of *S* from eastern Senegal had the same haplotype [34]. Assuming that selection for *AS* was 0.152 higher than for *AA,* the estimated age of this *S* allele was 1,440 years (see Table 1) [34]. Assuming that this allele was in low frequency (standing variation), it is likely that this age indicates the number of years since selection started to increase this allele.

Overall, there might have been five different mutations originating the *S* allele from the *A* allele. This conclusion is based on the presence of these variants in almost non-overlapping geographic regions, one found in both India and Saudi Arabia and four others in different parts of Africa, and the large differences in DNA sequence for genetic regions linked to *S*, resulting in different haplotypes in different geographic regions. The Mediterranean *S* alleles appear related to the African *S* haplotypes, particularly the Benin haplotype [14]. Recent parallel adaptation of different *S* alleles is consistent with an encompassing population genetics model [35].

In addition, Table 1 gives the estimated or assumed selection coefficient for different variants. For example, a selection coefficient of 0.152 for *S* indicates that the sickle-cell heterozygote *AS* has a 15.2% advantage over the normal homozygote *AA*. As a comparison, a selective advantage of more than a few per cent is unusual for other human genes.

A joint estimate of the age of the *C* allele and the selection coefficient was approximated with a coalescent approach [36]. The allele age was estimated to be 2,810 years and a selection coefficient of 0.06. More than one mutational origin for the *E* allele in Thailand is indicated [37]. On the other hand, the Chinese *E* allele has the same haplotype as in Thailand [38], implying the same origin. The age of the *E* allele was estimated to be about 2,510 years [39] and selection of 0.079.

Different ways have been used to estimate the age of G6PD mutants (Table 1). Using simulations, estimation of the age and selection for the *A*- allele was 6,360 years with selection of 0.044 and for the *Med* allele was 3,330 years and selection of 0.034 [40]. Using Bayesian methods, the estimated age of *A*- was only 1,000 years and selection was 0.25 [41] and the age of the *Mahidol* allele was 1,580 years with selection of 0.23 [42]. These estimates may differ because those in [40] used microsatellite loci and those in [41] used sequence data.

The age of the *HLA-Bw53* variant can be estimated using the approach below to estimate *s* (with *OR* = 0.59, *m* = 0.1, so that *s* = 0.041) and iteration of population genetic equations. Therefore, it would take approximately 2,150 years for *Bw53* to reach 0.25 [6]. This time is substantially less than the time that strong selection in Africa for malaria resistance is thought to have occurred.

There are several other malaria resistant variants that appear to be recent but for which there has been no formal estimation of their age. For example, out of about 80 deletions that result in *α*^+^ thalassaemia, four common ones have different geographic distributions; for example, deletion –*α*^3.7^ is found nearly worldwide but is most common in Africa, deletion –*α*^4.2^ has a high frequency in southeast Asia, and – *α*^3.7I^ is found most commonly in African, Indian and Mediterranean populations [14]. In addition, there are a number of mutants that have very limited geographic distributions. The age of these mutants has not been estimated, but their narrow distributions indicate recent generation.

Most of the *β*-thalassaemias are the result of single nucleotide substitutions or small insertions or deletions. Around 200 *β*-thalassaemias variants are known and they also have narrow geographic distributions suggesting that these changes indicate independent mutations and have been recently generated. *β*-thalassaemia variants in carriers are in frequencies from 5 to 20%, somewhat lower in frequency than *α*- thalassaemia variants [43].

A mutation in gene *SLC4A1* (erythrocyte Band 3 protein gene) found in Papua New Guinea and Malaysia results from a 27-nucleotide (9-amino acid) deletion. This genetic region is conserved throughout species [44] and the mutant causes abnormal ovalocytosis, an atypical red blood cell shape, and anaemia in heterozygotes. Even in progeny from matings between heterozygotes, homozygotes have not observed, indicating they are lethal [45]. The lethality of homozygotes and the high resistance of heterozygotes to malaria suggest that this variant has properties not very different from the *S* allele because the advantage to heterozygotes in resistance offsets the cost in fitness in homozygotes. Because of its limited distribution, it is thought to be a recent mutation.

There are several variants that provide resistance to malaria that appear to be older. For example, there are two antigens A and B produced by alleles FY*A and FY*B at the Duffy blood group locus [46,47]. Many African-Americans and Africans do not have either allele and have another allele, known generally as a Duffy null allele. This allele, FY*B^*ES*^, is in very high frequency in most of Africa and provides resistance to malaria species *Plasmodium vivax*, but not to *P. falciparum*[48].

The estimated age of the FY*B^*ES*^ allele, based on sequence analysis, is around 33,000 years [49], while the estimated age of this allele using microsatellite data is around 10,000 years ago [50]. The earlier estimated origin may be more consistent with the early impact of *P. vivax* on human mortality. Surprisingly, a Duffy allele in low frequency (0.022) in Papua New Guinea, also differs from FY*A in the same exact sequence as does the FY*B^*ES*^ allele found in Africa [51]. The low frequency of this allele is probably because of its recent origin, a situation seen in the pattern of alleles at closely linked loci [51].

### Examples of strong selection and change in allele frequency

As illustration, here two examples are given of fast expected genetic changes over time due to strong selection at genes conferring resistance to malaria. Both examples are ones in which there are two resistant variants in one population, one in which the genetic variants are different alleles at the same gene (*S* and *C*) and the other where the genetic variants are different alleles at different genes but there is epistasis between these variants (*S* and *α*-thalassaemia). Before these examples are described, an approach will be briefly introduced to estimate the extent of selection at these variants using contemporary case–control data (see [6] for details).

### Estimation of selection

A general approach to determine the risk of individuals with a given genotype getting a disease, relative to that in the rest of the population, is to calculate the odds ratio (*OR*) as *OR* = *f*_*d*_(1 – *f*_*c*_)]/*f*_*c*_(1 - *f*_*d*_)] where the frequencies of the genotype in control and diseased groups are *f*_*c*_ and *f*_*d*_, respectively. For example, the selection coefficient for the *AS* heterozygote can be estimated as *s* = *m*(1 – *OR*) where *m* is the rate of non-genotype specific mortality from malaria (generally the mortality given that an individual has severe malaria) [52,53].

To illustrate how *OR* and *s* can be calculated, data from a large study of malaria in The Gambia can be used to determine the effect of sickle-cell variation on the presence of severe malaria [54]. In 619 children with severe malaria, only seven (0.012) were heterozygous *AS*. On the other hand, in 510 other children, who were outpatients and termed “mild controls”, many more 66 (0.129) were *AS*. Therefore, *OR* = 0.082, indicating very great protection from severe malaria for *AS* genotypes. A value of 0.07 for *m* was suggested [52] while, a value of at least *m* = 0.1 may be appropriate [10,55]. For the *AS* genotype, if *OR* = 0.082 and *m* = 0.1, then *s* = 0.092.

### Increase of allele *C* in the presence of allele *S*

The polymorphic structural variants at the *β*-globin locus, *S* and *C*, both are malaria resistance alleles. Variant *S* is more widespread than *C,* which is in sub-Saharan Africa, but they both occur in a number of populations. Although some early studies suggested a stable polymorphism of these variants, a Burkina Faso sample [56] indicated the *C* allele would become fixed over time [53,57].

In the Burkina Faso sample, the genotypes *AC*, *AS*, and *CC* showed relative resistance to malaria and *AA* showed relative susceptibility [56]. The frequencies of these genotypes are given in Table 2 in both control and disease groups along with *OR* values. Fitnesses for genotypes *SC* and *SS* were based on deviations from Hardy-Weinberg proportions [53]. If *m* = 0.1, the relative fitness are given at the bottom in Table 2.

**Table 2:** **Frequency of different genotypes in healthy (control) subjects (*f***_***c***_**) and in malaria (diseased) patients (*f***_***d***_**) [**[56]**]**

			**Genotype**			
	***AA***	***AC***	***AS***	***CC***	***SC***	***SS***
*f*_*c*_	0.664	0.217	0.095	0.016	---	--
*f*_*d*_	0.804	0.164	0.028	0.001		
*OR*	2.070	0.708	0.268	0.072	---	---
Relative fitness	0.95	1.03	1.08	1.10	---	---
	0.86	0.94	0.98	1	0.50	0.11

The *OR* values for the genotypes and the estimated relative fitnesses are given (*m* = 0.1). In the bottom row, these values are standardized by the fitness of genotype *CC*, the genotype with the highest fitness [53].

**Figure 1:**
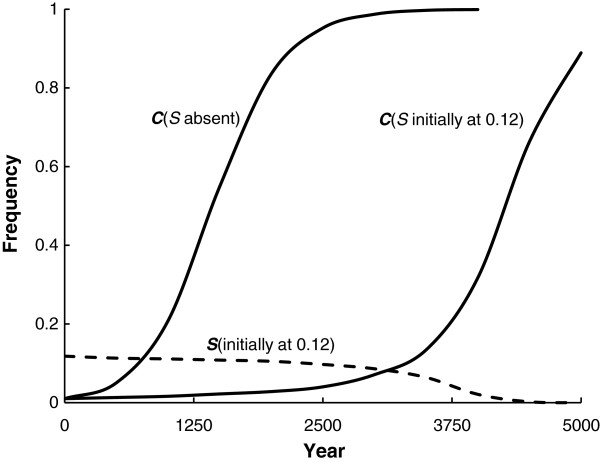
**The increase in frequency of allele *C *when it begins at a frequency of 0.01 (solid lines) both when *S *is absent and when *S *begins at its equilibrium frequency of 0.12 [ **[53]**].** The change in frequency of *S * is also given for the last situation (broken line).

With these fitnesses, when *S* is introduced to a population with only the *A* allele, it will go to a stable equilibrium with the frequency of *S* = 0.12. When *C* is introduced to a population with only the *A* allele, it will increase in frequency to fixation in a little more than 2,500 years (Figure 1). When the *S* and *A* alleles are at their stable equilibrium, then *C* can enter the population and eventually increase to fixation after about 5,000 years (Figure 1).

These results occur because genotype *CC* has the highest relative fitness of the genotypes. In addition, it might clarify situations, such as in the Dogon people of Mali who have a high frequency of *C* and a low *S* frequency [58]. In addition, and of public health consequence, the mean fitness of the population is about 11% higher when fixed for *C* than when it is polymorphic for *A* and *S* because of the absence of sickle-cell anaemia and higher resistance to malaria.

### Effect of epistasis between alleles *S* and *α*^*+*^*-*thalassaemia

Sickle cell anaemia and *α*^+^-thalassaemia both occur in some sub-Saharan African populations. The adult haemoglobin molecule is composed for two subunits of each of the *α*-globin and *β*-globin molecules. As a result, it is not unexpected that there could be gene-gene interaction, or epistasis, at these loci for fitness. In a Kenyan population, these disorders were both present [59] and when the two disorders were inherited together in the same individual, the protection from malaria given individually by each allele was lost (see also [60]). This interaction, or negative epistasis, could be part of the explanation why *α*^+^-thalassaemia does not appear fixed in sub-Saharan populations. A traditional population genetics approach is used here to examine this situation, for an epidemiological approach, see [59,61,62]

The relative viabilities (fitnesses) of the different two-locus genotypes (Table 3) were based on genotype-specific annual mortality rates (see details in [6,59]). With a wild-type genotype at both genes, the estimated relative fitness is only 0.55. The presence of *S* in *AS* and the presence of −*α* in both genotypes −*α*/*αα* and −*α*/−*α* increases the estimated relative fitness, given normal genotypes at the other locus. However, the relative survival is reduced to only 0.55 when there is an *AS* genotype and homozygosity for −*α*, the same as the relative fitness for the wild-type genotype.

**Table 3:** **Estimated relative survival (fitness) values for the two-locus genotypes based on the data from**[59]**(see**[6]**for details)**

	***α*-globin genotype**		
***β*- globin genotype**	***αα/αα***	**−*a/αα***	**−*α/−α***
*AA*	0.55	0.68	0.70
*AS*	1	0.91	0.55
*SS*	0	0	0

This array of relative fitness values illustrates several things. First, there is a stable polymorphism with *S* equal to 0.309 when only the normal *αα* haplotype at the *a*-globin gene is present (first column). Second, variant –*α* haplotype would be fixed when only the normal *A* allele is present at the *β*-globin locus (first row) because the –*α/*−*α* genotype has the highest fitness. In addition, some two-locus behaviour can be intuited by looking at these viability values further. For example, if the population is fixed for –*α/*–*α* it does not appear that *S* can enter the population because *AA* –*α*/−*α* >*AS* −*α*/−*α* (Table 3).

**Figure 2:**
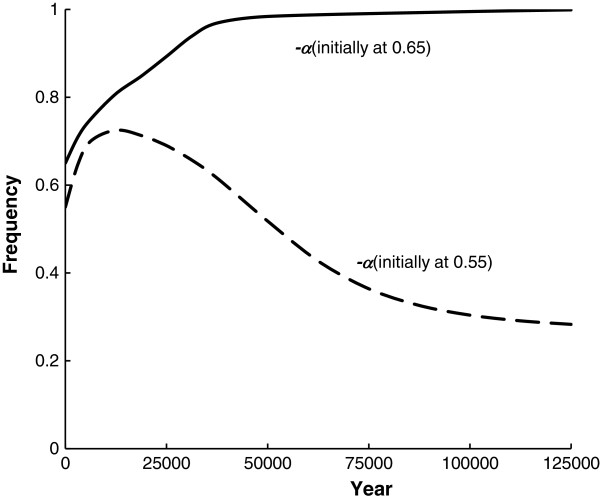
**The change in the frequency of – *a *where the initial frequency is either 0.55 or 0.65 using the relative fitnesses in Table  **3**and the initial frequency of *S *is 0.01. **

Using iterations of two-locus gamete frequency equations with selection [63], more details of the genetic behaviour of gametes with this two-locus fitness array are observed. For example, if –*α* is polymorphic, there is an unstable equilibrium so that –*α* will either increase or go to a two-locus equilibrium, depending upon its initial frequency, if *S* is introduced into the population. Figure 2 presents this situation where the initial frequency of *S* is 0.01 and the initial frequency of –*α* is either 0.55 or 0.65. The frequency of –*α* initially increases for 0.55 and then approaches the two-locus equilibrium at 0.272. On the other hand, when the initial frequency is 0.65, it increases to unity while *S* initially increases but then decreases to 0 (for further discussion see [6]). Notice that although the amount of epistasis between the genes is high, the time for these changes to occur is quite long.

## Conclusions

Obviously, Haldane [3] was correct to point out that malaria resistance in humans was a significant evolutionary factor. Malaria resistance genes comprise some of the most widely accepted examples of strong positive selection in humans. Overall, the original “malaria hypothesis” of Haldane that diseases like thalassaemia are polymorphisms because of resistance to malaria, has been proven correct. However, much is still to be learned about actual mechanisms of protection, other genes that confer resistance, and the population genetics of this variation.

Malaria resistance offers cases of a number of aspects of population genetics, particularly situations of recent, strong selection. Overall, many of these resistance variants appear less than 5,000 years old, much more recent than for most human variants. They also have selection coefficients significantly larger than 1%, much stronger than for most human variants. Population genetics will probably be even more important in understanding the joint impact of multiple malaria resistance variants in the future. Two situations are examined here, and it is shown that in populations segregating for *S* and *C*, selection is expected to eliminate *S* and fix *C*. Also, depending upon the starting frequencies, in populations segregating for *α*-thalassaemia and *S*, either the *α* -thalassaemia haplotype will become fixed and the *S* allele eliminated or a stable equilibrium of both variants will occur because of negative epistasis.

## Competing interests

The author declares there are no competing interests.

## Authors’ contribution

PWH conceived and wrote this manuscript. The author read and approved the final manuscript.
